# Elevated levels of adaption in *Helicobacter pylori* genomes from Japan; a link to higher incidences of gastric cancer?

**DOI:** 10.1093/emph/eov005

**Published:** 2015-03-18

**Authors:** Maria Juliana Soto-Girón, Oscar E. Ospina, Steven Edward Massey

**Affiliations:** Bioinformatics Lab, Department of Biology, University of Puerto Rico - Rio Piedras, PO Box 23360, San Juan 00931, Puerto Rico

**Keywords:** *Helicobacter pylori*, positive selection, pathogenic factors, PAML, pairwise comparison, Ka/Ks

## Abstract

*Helicobacter pylori* is a bacterium that lives in the human stomach and is a major risk factor for gastric cancer and ulcers. *H.pylori* is host dependent and has been carried with human populations around the world after their departure from Africa. We wished to investigate how *H.pylori* has coevolved with its host during that time, focusing on strains from Japanese and European populations, given that gastric cancer incidence is high in Japanese populations, while low in European. A positive selection analysis of eight *H.pylori* genomes was conducted, using maximum likelihood based pairwise comparisons in order to maximize the number of strain-specific genes included in the study. Using the genic Ka/Ks ratio, comparisons of four Japanese *H.pylori* genomes suggests 25–34 genes under positive selection, while four European *H.pylori* genomes suggests 16–21 genes; few of the genes identified were in common between lineages. Of the identified genes which were annotated, 38% possessed homologs associated with pathogenicity and / or host adaptation, consistent with their involvement in a coevolutionary ‘arms race’ with the host. Given the efficacy of identifying host interaction factors *de novo*, in the absence of functionally annotated homologs our evolutionary approach may have value in identifying novel genes which *H.pylori* employs to interact with the human gut environment. In addition, the larger number of genes inferred as being under positive selection in Japanese strains compared to European implies a stronger overall adaptive pressure, potentially resulting from an elevated immune response which may be linked to increased inflammation, an initial stage in the development of gastric cancer.

## Introduction

*Helicobacter pylori* is a Gram negative bacterium that colonizes the human stomach and is adapted to its harsh acidic conditions to such an extent that it is completely dependent on its host. The majority of people around the world are infected with *H.pylori*, although infection rates may vary according to the region [1]. *H.pylori* is often pathogenic, with infection linked to gastric cancer and ulcers [2]. Gastric cancer is the second most common cause of death from cancer worldwide, with Korea, Japan and China demonstrating the highest incidences [3]. In contrast, European populations have a low incidence of both gastric cancer and ulcers [4]. While *H.pylori* infection is a major risk factor for gastric cancer [5], not all epidemiological studies have shown a direct relationship between *H.pylori* infection and gastric cancer [6]. These inconsistencies may be due in part to additional factors such as smoking, alcohol consumption and diet, variations in the immune response, as well as the *H.pylori* genotype [7, 8]. It is also notable that the incidence of gastric cancer has fallen over the past few decades in Europe [9] from a 19th century high in some countries [10, 11], and this reduction has been attributed to a variety of factors such as a drop in smoking, changes in diet and possibly a reduction in *H.pylori* infection [12]. Interestingly, there is growing evidence that *H.pylori* may be beneficial early in life, protecting from acid-related esophageal diseases [13–15], asthma [16, 17], and potentially obesity [18]. Thus, the effects of *H.pylori* infection on the host appear more complex than simply a host–pathogen interaction.

The *H.pylori* genotype may predispose an individual towards the development of gastric cancer if certain pathogenic factors are present [2]. One such factor is the *cag* pathogenicity island (*cag* PaI), which encodes a Type IV secretion system that injects the oncogenic CagA protein into host cells [19]. Strains that possess it are linked with an elevated incidence of gastric cancer and ulcers [2]. In East Asian countries, elevated gastric cancer rates are linked to the presence *H.pylori* strains that possess CagA with an EPIYA-D motif [20]. Another gene linked to regional variation in gastric cancer incidence is the cytotoxin VacA. The *vacA s1m1* genotype predominates in Japan and Korea and has been linked to a higher incidence of gastric cancer, compared to the *vacA s2m2* genotype that is more common in the West [20].

Additional genes associated with pathogenicity in *H.pylori* include the blood group antigen binding and sialic acid-binding adhesin genes [*babA* and *sabA;* 21, 22], the *iceA* gene [23], outer inflammatory protein and duodenal ulcer promoting genes [*oipA* and *dupA*; 24, 25] and flagellin subunit A gene [*flaA;* 26]. *babA*, *sabA* and *iceA* are involved in cell adhesion, *oipA* and *dupA* are involved in the induction of inflammation and *flaA* is involved in motility. Given that *H.pylori* varies substantially in strain-specific genes [27], the possibility exists that there are additional unidentified genes associated with pathogenicity and that these are partly responsible for regional differences in gastric cancer and ulcer incidences.

In pathogen genomes, a proportion of genes under positive selection are expected to be pathogenic factors. This is because pathogenic factors are involved in a coevolutionary ‘arms race’ with the host, particularly those interacting directly with the immune system and other host receptor proteins. Competition with the host leads to rapid adaptation and signatures of positive selection [28]. In addition, a role in pathogenicity may be indicated by involvement on the cell surface, where many pathogenic factors have a role in cell adhesion, toxin secretion and host recognition [29]. Here, in an effort to identify novel pathogenic factors in the *H.pylori* genome, and strain-specific differences in overall adaptive pressure which might be linked to differences in the host immune response, we used a maximum likelihood Ka/Ks-based method to infer which genes were under positive selection.

## Methods

### Data source and phylogenetic analysis

**Figure 1.**
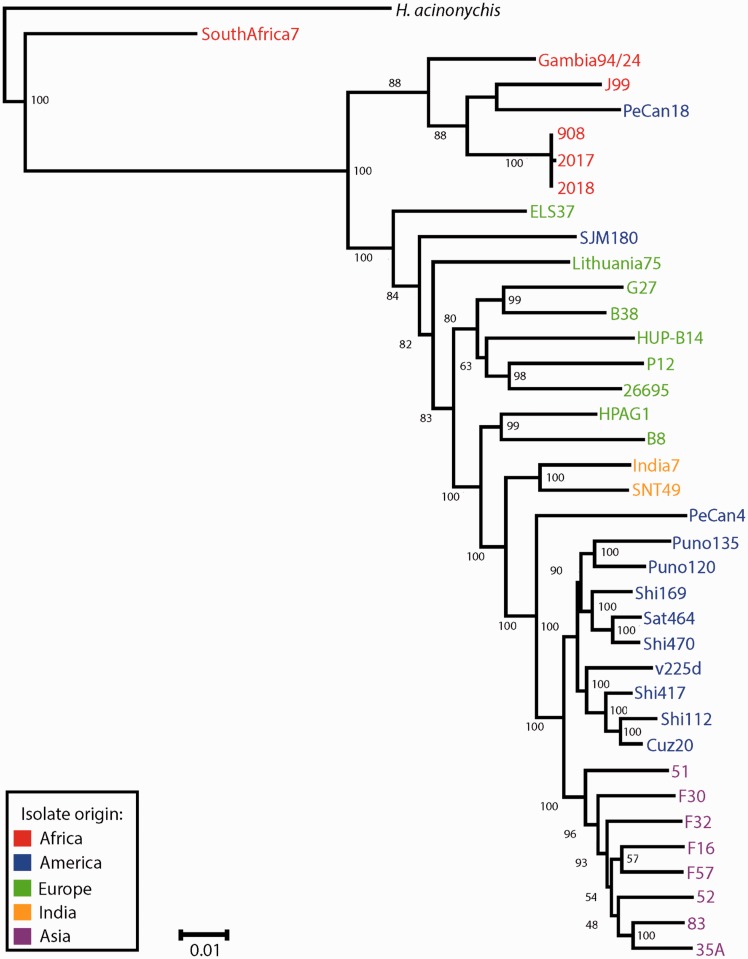
Phylogenetic analysis of 37 *H.pylori* strains with complete genomes. Bayesian phylogenetic inference of 37 *H.pylori* strains was conducted as described in Methods. Colors indicate the region from which each strain was isolated. Numbers at nodes represent posterior probabilities

A phylogenetic tree of 37 *H.pylori* strains with complete genomes available was constructed (Fig. 1). The strains, with NCBI Refseq ID, country of origin and associated disease status where available (Ga; gastritis, PUD; peptic ulcer disease, ML; MALT lymphoma, GC; gastric cancer, C; commensal) were as follows. SouthAfrica7 (NC_017361.1; South Africa; C), Gambia94/24 (NC_017371.1; Gambia; C), J99 (NC_000921.1; USA; PUD), PeCan18 (NC_017742; Peru; GC), 908 (NC_017357.1; West Africa; PUD), 2017 (NC_017374.1; West Africa; PUD), 2018 (NC_017381.1; West Africa; PUD), ELS37 (NC_017063.1; El Salvador; GC), SJM180 (NC_014560.1; Peru; Ga), Lithuania75 (NC_017362.1; Lithuania; C), G27 (NC_011333.1; Italy; PUD), B38 (NC_012973.1; France; ML), HUP-B14 (NC_017733.1, Spain; C), P12 (NC_011498.1, Germany, PUD), 26695 (NC_000915.1; UK; Ga), HPAG1 (NC_008086.1; Sweden; Ga), B8 (NC_014256.1; USA; PUD), India7 (NC_017372.1; India; PUD), SNT49 (NC_017376.1; India; C), PeCan4 (NC_014555.1; Peru; GC), Puno135 (NC_017379.1; Peru; Ga), Puno120 (NC_017378.1; Peru; Ga), Shi169 (NC_017740.1; Peru; C), Sat464 (NC_017359.1; Peru; C), Shi470 (NC_010698.2; Peru; Ga), v225d (NC_017355.1; Venezuela; Ga), Shi417 (NC_017739.1; Peru; C), Shi112 (NC_017741.1; Peru; C), Cuz20 (NC_017358.1; Peru; C), 51 (NC_017382.1; South Korea; PUD), F30 (NC_017365.1; Japan; PUD), F32 (NC_017366.1; Japan; GC), F16 (NC_017368.1; Japan; Ga), F57 (NC_017367.1; Japan; GC), 52 (NC_017354.1; South Korea), 83 (NC_017375.1; Japan; GC), 35A (NC_017360.1: Japan; PUD). Sequences from the *H.acinonychis* genome (NC_008229.1) were used as an outgroup.

The phylogenetic tree was constructed as follows. The housekeeping genes *atpA*, *atpD*, *efp*, *glnA*, *mutY*, *ppa*, *trpC*, *ureI* and *yhpC* were obtained from each genome, concatenated, and a multiple alignment constructed using the Muscle program [30] and default parameters. This was used as input to the JModelTest program [31] to identify an appropriate substitution model for the phylogenetic analysis. The best model selected using the Akaike Information Criterion was GTR + I + Г, with values of I = 0.638 and Г = 0.454. Bayesian phylogenetic inference was conducted using the MrBayes 3.2 program [32], running the analysis for 8 000 000 generations and discarding as burn-in the first 25% of the samples generated.

### Genome-wide positive selection pipeline

Pairwise genome comparisons, using four European and four Asian *H.pylori* strains were conducted, using a third strain as an outgroup in each comparison. The maximum likelihood-based branches test of PAML (Phylogenetic Analysis by Maximum Likelihood) package version 4 [33] was utilized for the comparisons, which produces an estimate of the genic Ka/Ks ratio, which is calculated over the entire length of the protein coding gene. Because of this, the genic Ka/Ks ratio is a strong indicator of positive selection when detected [34, a review]. A value of Ka/Ks > 1 is consistent with positive selection, Ka/Ks < 1 is consistent with negative selection and Ka/Ks = 1 is consistent with an absence of selection (neutrality). The maximum likelihood approach that we follow is more accurate than the commonly used ‘approximate’ methods to calculate the genic Ka/Ks [35–38], and also has the advantage of giving lineage specific information. The alternative branches sites test [39] looks for individual sites that may be under positive selection, using the Ka/Ks ratio at each site. While more sensitive at detecting lineage specific positive selection, it is also prone to false positives resulting from sequencing and alignment errors [40–42], the effects of recombination [43, 44], and segregating polymorphisms [45]. Lastly, for pairwise comparisons its use is inappropriate given that substantial numbers of orthologous sequences are required for the alignment [33, 46] and this necessitates a known phylogeny with 100 % certainty [47, a review], which is not available for *H.pylori.* Lastly, more limited pairwise comparisons allow the analysis of strain-specific genes that would be excluded if the *H.pylori* core genome of larger numbers of strains were used, given that known pathogenic factors are often strain specific in *H.pylori.*

Pairs of *H.pylori* strains were selected using the phylogenetic tree as a basis; those pairs that formed monophyletic groups were utilized. For the European strains these were B38/G27, and 26695/p12. For the Asian strains these were 35A/83 and F16/F57. The Asian strains were all from Japanese populations; 35A and 83 isolated from individuals from Kyoto, F16 and F57 were isolated from individuals from Fukui (Yoshio Yamaoka, Michael E. DeBakey Veterans Affairs Medical Center, Baylor College of Medicine, personal communication). The HUP-B14 strain was used as an outgroup for both European pairwise comparisons and strain 52 was used for both Japanese pairwise comparisons. All pairs chosen were statistically supported (posterior probability >0.95), with the exception of the F16/F57 pair, which were statistically superior to the alternative F16/F32 and F16/F52 pairs (Fig. 1). In this case, while the use of alternative pairs would not affect the nature of the analysis, their choice would be arbitrary and we wished to use the most likely monophyletic pair according to the phylogenetic analysis. Because an unrooted tree is used for the analysis, the phylogenetic relationship of the pair to the outgroup is not expected to affect the outcome, and this is expected to minimize the potential effects of recombination given that the sequences are equidistant phylogenetically.

**Figure 2.**
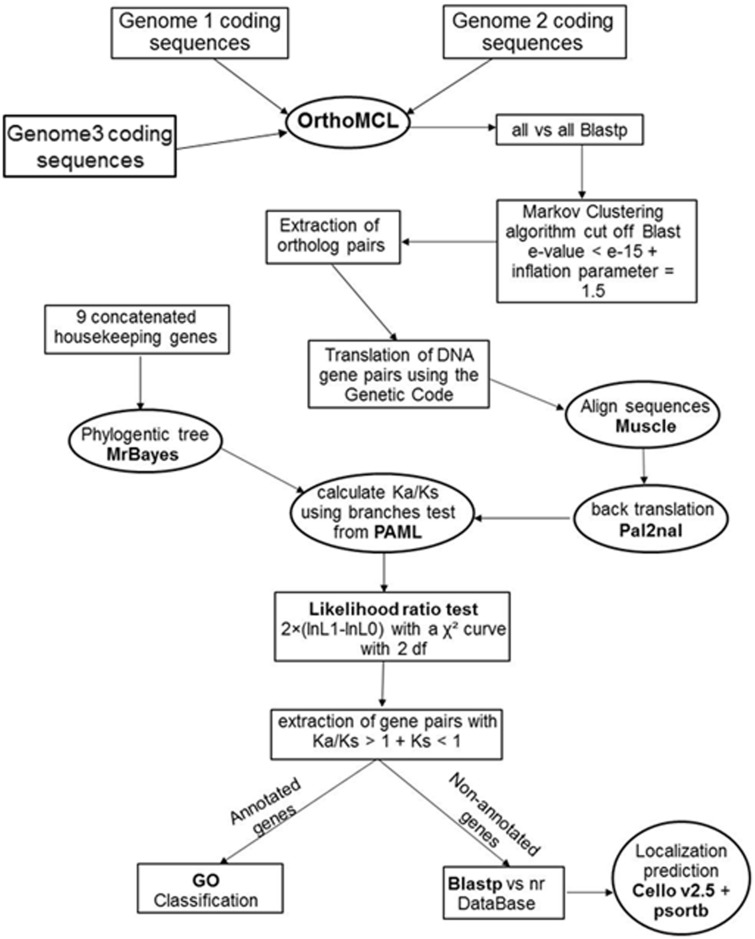
Pipeline for the inference of genes under positive selection in pairwise genome comparisons. Schematic representation of the selection analysis pipeline showing each step of a pairwise genome comparison from identification and extraction of the ortholog pairs to the functional categorization of the proteins under inferred positive selection

A pipeline was developed using Python, integrating a variety of different software packages (Fig. 2); the script is available from the authors upon request. The first step of the analysis was to identify all orthologous genes present in each set of three strains (pairwise comparison and outgroup). This was accomplished using the OrthoMCL program [48]. OrthoMCL begins with a reciprocal all-against-all BLASTP search within strains, to identify putative paralogs, and between strains, to identify putative orthologs. The default cutoff e-value of 1e-5 was used. Putative paralogous and orthologous relationships are converted into a graph, which is then subjected to the MCL algorithm [49] in order to identify orthologous groups. An inflation value of 1.5 (default) was used for this stage of the analysis. The output was used to construct multiple alignments of orthologous protein sequences for each set of strains, using Muscle and default parameters. Then, each alignment was converted to DNA using the PAL2NAL program [50] and the respective DNA sequence corresponding to each protein sequence. This procedure ensures the correct placement of gaps.

To infer genes under positive selection, the Ka/Ks (or ω) ratio was calculated using the branches test of the codeml module of PAML [33], for each set of orthologous genes. The null model was the one ratio model, where the value of ω is fixed, while the alternative model allows the value of ω to vary for individual branches (free ratio test). Likelihood values were generated for both models and a likelihood ratio test was applied using a Χ^2^ distribution to calculate *P* values. Those gene comparisons that were not significant (*P* > 0.05) were excluded from further analysis. Values of Ka, Ks and Ka/Ks were extracted for each gene pair. Saturation of synonymous substitutions can lead to an underestimate of Ks and thus an overestimate of Ka/Ks [51]. Synonymous site saturation was considered as Ks > 1 (which means that all sites have been substituted at least once on average), and sequences with this value were excluded from the analysis. Given that there were some differences in branch lengths between the European and Japanese strains (Fig. 1), analyses that estimated genes with Ks = 0 were excluded, given that this may lead to an inflated value of Ka/Ks. Genes under positive selection were inferred with a value of Ka/Ks > 1 and *P* < 0.05. A *P* value < 0.05 has been shown in simulation studies to reliably minimize the occurrence of false positives, while maintaining the power of the maximum likelihood approach [52], which is important given that the genic Ka/Ks ratio is a conservative test for positive selection.

### Functional characterization

Genes inferred to be under positive selection which possessed a functional assignment from the genome annotation, were categorized using their gene ontology (GO) classifications [53]. GO enrichment was conducted using Blast2Go [54]. Unannotated genes (identified as ‘hypothetical proteins’) were blast searched against the non-redundant Genbank database. If they possessed significant sequence similarity with proteins of known function (over 30% sequence identity at the amino acid level and e-value < e − 15), then they were classified as homologs. Genes with no known homologs that were inferred as being under positive selection were subject to localization prediction in order to infer whether they are membrane localized. The CELLO v.2.5 program [55] and PSORTb v.3.0 program [56] were used to predict the membrane localization of the proteins.

## Results and Discussion

The complete genome sequences of the eight strains chosen for this study were aligned and show the recombinergic nature of the *H.pylori* genome, as previously noted [57] (Supplementary Fig. S1). Table 1 presents statistics from the eight pairwise genome comparisons. The numbers of genes analyzed after filtering are similar, with the 26695/P12 comparison presenting the highest number of gene pairs (1247), and the 35a/83 comparison the least (1173). The genome pairs were closely related to each other, with average lineage specific Ks values ranging from 0.023 substitutions per site (strain 35a) to 0.058 substitutions per site (strain 26695). Thus, the level of divergence between genome pairs was such that a substantial proportion of the genes in each genome were included in the analysis after filtering, which removes saturated and non-divergent sequences. The similarity in the numbers of genes retained is important for comparing the results of the different comparisons.

**Table 1.** Statistics from the four pairwise genome comparisons of *H.pylori* strains from Japan and Europe

Pairs	Strain (geographic origin in brackets)	Total genes present in genome	Genes pairs remaining after filtering	Average Ks (substitutions per site)	Average Ka/Ks < 1	Number of genes under inferred positive selection	Percentage of total genes
Japan1	F57 (Fukui)	1563	1191	0.034	0.11	31	1.98
	F16 (Fukui)	1543		0.030	0.11	30	1.94
Japan2	35a (Kyoto)	1560	1173	0.023	0.10	25	1.60
	83 (Kyoto)	1656		0.031	0.10	34	2.10
Europe1	G27 (Italy)	1581	1181	0.054	0.15	17	1.08
	B38 (France)	1571		0.051	0.16	16	1.02
Europe2	P12 (Germany)	1634	1247	0.054	0.16	21	1.29
	26695 (UK)	1563		0.058	0.17	16	1.02

Overall, the majority of the genes examined from the eight comparisons had Ka/Ks < 1, consistent with the expectation that the majority of the genes in a genome should be under purifying selection. The average Ka/Ks for each genome was calculated only from these genes, with values ranging from 0.10 (strains 35a and 83) to 0.17 (strain 26695; Table 1). These values indicate that the genomes are under similar evolutionary pressures in different human populations regarding purifying selection, and imply that modes of transmission, host genetics and environmental factors do not exert a significant influence on the strength of purifying selection.

**Figure 3.**
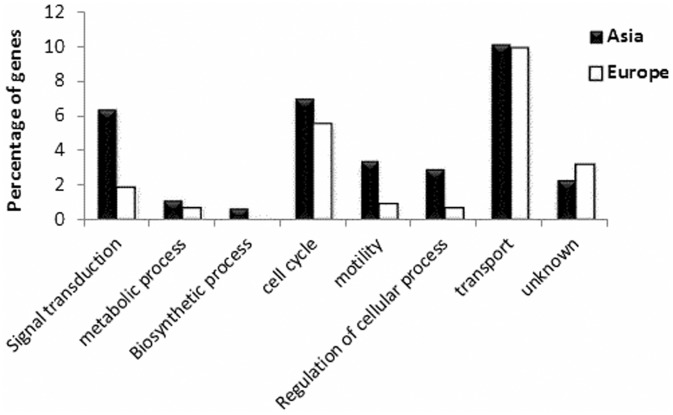
Gene Ontology enrichment of genes under inferred positive selection. Functional categories for all genes with Ka/Ks > 1 of the pairwise comparisons from Europe (B38 vs G27 and HPAG1 vs 26695) and Japan (F16 vs F57 and 35a vs 83) pairwise comparisons, using the Gene Ontology Database and Blast2Go, described in Methods. The numbers displayed on the y-axis represent the relative enrichment in terms of the proportion of positively selected genes in each GO category compared to the total number present in that category in the genome

Positive selection was inferred to be acting on a number of protein coding genes from all *H.pylori* pairwise comparisons. The Japanese strain 83 presented the highest number of genes with Ka/Ks > 1 (34 genes), while the European strains B38 and 26695 had the least, with 16 genes each. Genes inferred as being under positive selection were significantly elevated in Japanese strains compared to European strains (*P* < 0.05, two-tailed Mann–Whitney test). Thus, the numbers of genes inferred as being under positive selection in the *H.pylori* strains may vary by a factor of 2. Strain B38 possesses a phage present in the genome [58], but none of the genes inferred as being under positive selection were phage associated. GO enrichment shows that transport proteins are particularly highly represented, reflecting a major role for membrane proteins in adaptation (Fig. 3).

**Figure 4.**
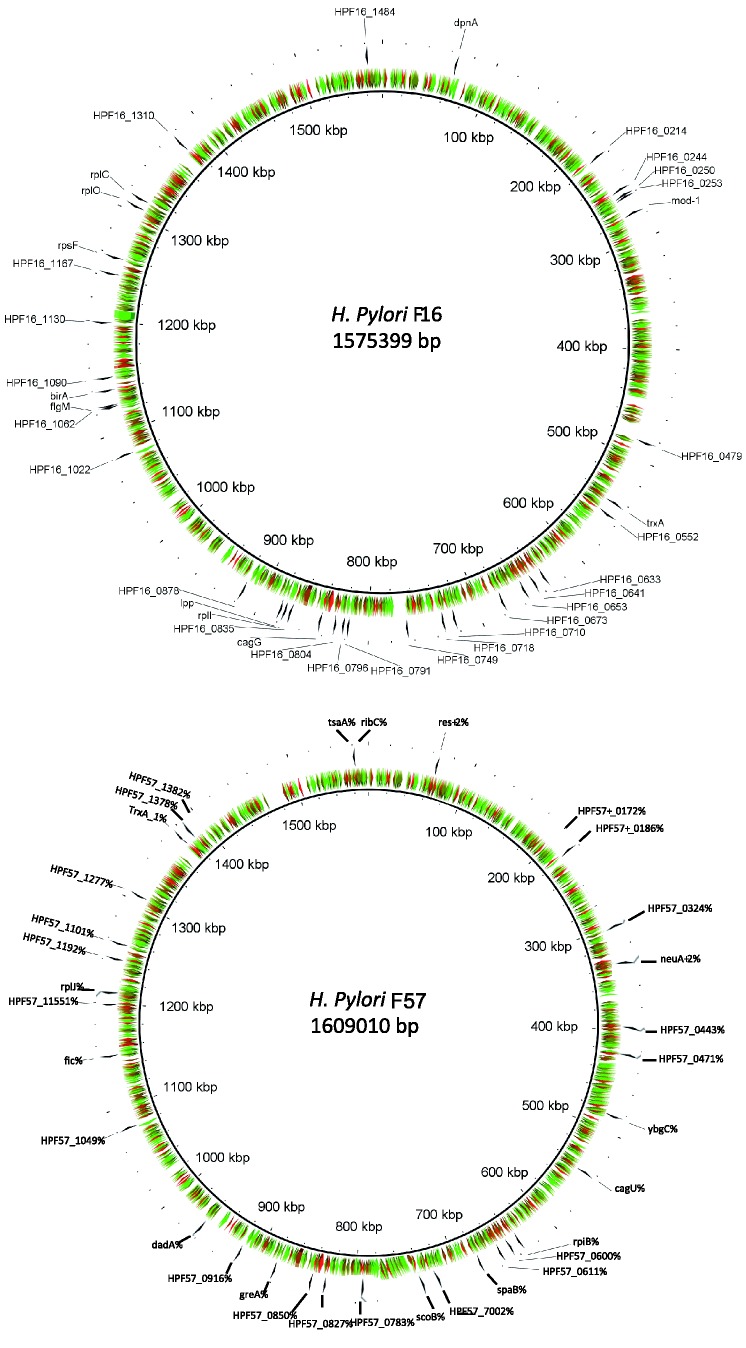
Selection map of *H.pylori* strains F16 and F57. Patterns of selection were plotted around the genomes of *H.pylori* strains F16 and F57, isolated from patients from Fukui, Japan. Genes inferred as being under positive selection are indicated with arrows. Colors represent the strength of purifying selection, with the scale ranging from Ka/Ks = 0 (red) to Ka/Ks = 0.4 (green).

Tables 2 and 3 list those genes inferred to be under positive selection for the four comparisons for Japan and Europe, respectively. A total of 190 genes were inferred to be under positive selection, of these 65 were unannotated (described as ‘hypothetical protein’ or ‘conserved hypothetical protein’). When the Ka/Ks values for different individual genes are plotted around the genome for strains F16 and F57 a mosaic pattern of selection values is observed, with no regions strongly associated with enhanced negative selection pressure (Fig. 4). However, strain F16 shows a number of distinct clusters of genes inferred as being under positive selection; these could be termed ‘islands of adaptation’. This clustering is not observed with strain F57, where the distribution appears largely random.

**Table 2.** Genes under inferred positive selection from the pairwise genome comparisons of four *H.pylori* strains isolated from Japan

35a (Kyoto, PUD)	83 (Kyoto, PUD)	F16 (Fukui, Ga)	F57 (Fukui, GC)
Flagellar exodeoxyribonuclease hook-associated protein 2, fliD (YP_005769952.1) (*P* value: 0.0001)	Lipid A 1-phosphatase (YP_005769383.1) (*P* value: 1.10E−05)	Urease-enhancing factor (YP_005779079) (*P* value: 1.15E−06)	Thioredoxin (BAJ55951.1) (*P* value: 0.001)
Probable outer membrane protein (ADU41365.1) (*P* value: 0.001)	DNA-directed RNA polymerase subunit omega (YP_005769931.1) (*P* value: 3.67E−05)	50S ribosomal protein L9 (BAJ55437.1) (*P* value: 3.94E−07)	Outer membrane protein, HorA (YP_005776806.1) (*P* value: 0.002)
Dethiobiotin synthetase (YP_005770624.1) (*P* value: 0.0001)	6,7-dimethyl-8-ribityllumazine synthase (riboflavin synthase beta chain) (YP_005769366.1) (*P* value: 0.001)	30s ribosomal protein S6 (BAJ55777.1) (*P* value: 0.00)	Conserved hypothetical ATP-binding protein (BAJ54919.1) (*P* value: 0.00)
Neuraminyllactose-binding hemagglutinin (YP_005769912.1) (*P* value: 0.00)	ATP-binding protein (YP_005770583.1) (*P* value: 0.0008)	50S ribosomal protein L3 (BAJ55847.1) (*P* value: 0.00)	Putative secretion/efflux ABC transporter ATP-binding protein (YP_006227497.1) (*P* value: 0.001)
Urease-enhancing factor (YP_005770181) (*P* value: 0.00)	Phosphonate metabolism protein PhnI (YP_005769292.1) (*P* value: 0.0002)	ABC transporter permease (BAJ55515.1) (*P* value: 0.00)	CMP-N-acetylneuraminic acid synthetase (BAJ54930.1) (*P* value: 0.001)
Periplasmic competence protein (YP_005769282.1) (*P* value: 0.00)	Type II methylase protein (YP_005769308.1) (*P* value: 1.03E−05)	Sialidase A (YP_003728769.1) (*P* value: 0.001)	Putative endonuclease (YP_005776737.1) (*P* value: 0.002)
gfo/Idh/MocA family oxidoreductase (YP_005770018.1) (*P* value: 0.0001)	Methyl-accepting chemotaxis protein (YP_005769452.1) (*P* value: 6.64E−05)	Type II modification enzyme (BAJ55149.1) (*P* value: 0.002)	D-Amino acid dehydrogenase (BAJ55519.1) (*P* value: 0.002)
Pseudouridine synthase D (YP_005769708.1) (*P* value: 0.0002)	Lipopolysaccharide ABC superfamily ATP binding cassette transporter permease protein (YP_005769347.1) (*P* value: 3.92E−15)	Adenine-specific DNA methyltransferase (BAJ54643.1) (*P* value: 0.001)	RNA-binding protein (BAJ55130.1) (*P* value: 0.001)
Ribosomal protein L11 methyltransferase (YP_005769743.1) (*P* value: 0.001)	Shikimate kinase (YP_005769533.1) (*P* value: 4.29E−06)	cag pathogenicity island protein (CagG) (BAJ55413.1) (*P* value: 0.002)	Putative lipopolysaccharide biosynthesis protein (BAJ55644.1) (*P* value: 0.00)
50S ribosomal protein L22 (YP_005770576.1) (*P* value: 0.00)	UDP-sugar diphosphatase (YP_005770185.1) (*P* value: 0.002)	flgM protein (BAJ55661.1) (*P* value: 0.001)	ABC transporter ATP-binding protein (YP_005777218.1) (*P* value: 0.001)
Holliday junction resolvase family protein (YP_005769700.1) (*P* value: 0.00)	NADH-quinone oxidoreductase subunit A (YP_005770524.1) (*P* value: 0.0004)	7-cyano-7-deazaguanine reductase (BAJ55907.1) (*P* value: 0.001)	cell filamentation protein (BAJ55696.1) (*P* value: 0.001)
Disulfide interchange protein (YP_005770340.1) (*P* value: 0.00)	Poly(A) polymerase (YP_005770052.1) (*P* value: 5.45E−05)	Biotin protein ligase (BAJ55679.1) (*P* value: 0.001)	Ribose-5-phosphate isomerase B (BAJ55382.1) (*P* value: 0.00)
Flagellar hook-basal body complex protein FliE (YP_005769337.1) (*P* value: 0.00)	Proteobacterial sortase system OmpA family protein (YP_005769939.1) (*P* value: 3.67E−05)	RNA-binding protein (BAJ55130.1) (*P* value: 0.00)	Peptidase M50 (YP_005770113.1) (*P* value: 0.00)
Orotate phosphoribosyltransferase (YP_005770521.1) (*P* value: 0.00)	DASS family divalent anion:sodium (Na+) symporter (YP_005769519.1) (*P* value: 0.0002)	Type II DNA modification enzyme (BAJ55625.1) (*P* value: 0.0003)	feoA gene product (YP_005770102.1) (*P* value: 0.00)
CDP-diacylglycerol-glycerol-3-phosphate, 3-phosphatidyltransferase (YP_005769796.1) (*P* value: 0.001)	Phosphoserine phosphatase (YP_005770041.1) (*P* value: 5.61E−05)	Type II restriction endonuclease (BAJ55626.1) (*P* value: 0.0003)	Riboflavin synthase subunit alpha (YP_005778223.1) (*P* value: 3.19E−05
Fatty acid/phospholipid synthesis protein PlsX (YP_005769572.1) (*P* value: 0.00)	Polar flagellin (YP_005769953.1) (*P* value: 0.0007)	Riboflavin synthase subunit alpha (YP_005779723.1) (*P* value: 2.14E−4)	Type II restriction endonuclease (YP_005777226.1) (*P* value: 3.59E−06)
Pyrroline-5-carboxylate reductase (YP_005770419) (*P* value: 0.00)	Chorismate mutase (YP_005769657.1) (*P* value: 4.39E−05)	Secreted protein involved in flagellar motility (YP_006219827.1) (*P* value: 0.003)	Transcription elongation factor GreA (BAJ55095.1) (*P* value: 0.001)
H. protein HPF30_0649 (ADU41023) (*P* value: 0.00)	50S ribosomal protein L15 (YP_005770563) (*P* value: 0.001)	H. protein HPF16_0633 (YP_005778868) (*P* value: 0.0007)	50S ribosomal protein L10 (YP_005777971.1) (*P* value: 5.91E−06)
H. protein HPCPY3281_0951 (ADU40935) (*P* value: 0.00)	Thiamine-phosphate diphosphorylase (YP_005769865) (*P* value: 5.97E−07)	H. protein HPF16_0710 * (YP_005778945) (*P* value: 0.0001)	Succinyl-CoA-transferase subunit B (BAJ55301.1) (*P* value: 0.001)
H. protein HPHPA14_0570 (ADU40948) (*P* value: 0.00)	HOP family outer membrane porin (YP_005769995) (*P* value: 2.35E−09)	H. protein HPF16_0214 (YP_005778449) (*P* value: 6.05E−05)	Biotin sulfoxide reductase BisC fragment (YP_005777721.1) (*P* value: 0.001)
Conserved hypothetical protein (ADU40534.1) (*P* value: 0.00)	Integral membrane protein (YP_005769244) (*P* value: 7.67E−06)	H. protein HPF16_0250 (YP_005778485) (*P* value: 0.001)	cag pathogenicity island protein (CagU) YP_005777275.1) (*P* value: 7.47E−06)
H. protein HP9810_881g5 (ADU40463) (*P* value: 0.002)	Undecaprenyl phosphate N-acetylglucosaminyltransferase (YP_005769359) (*P* value: 1.52E−08)	H. protein HPF16_0244 (YP_005778479) (*P* value: 0.002)	H. protein HPF57_1192 (YP_005777906.1) (*P* value: 0.006)
H. protein HP0385 (ADU41339) (*P* value: 0.00)	Acyl-phosphate glycerol 3-phosphate acyltransferase (YP_005769268) (*P* value: 2.25E−05)	H. protein HPF16_1359 * (BAJ55956) (*P* value: 0.001)	H. protein HPF57_0261 (YP_005776975.1) (*P* value: 1.88E−05)
H. protein HPCPY6311_1233 (ADU41477.1) (*P* value: 0.00)	Conserved hypothetical protein * (ADU41024) (*P* value: 8.6E−05)	H. protein HPF16_0796 (BAJ55393) (*P* value: 0.001)	H. protein HPF57_0394 (YP_005777108.1) (*P* value: 5.47E−12)
H. protein HPKB_0158 (ADU40191.1) (*P* value: 0.00)	Conserved hypothetical protein * (ADU41633) (*P* value: 0.01)	H. protein HPF16_0423 * (BAJ55020) (*P* value: 0.001)	H. protein HPF57_0426 (YP_005777140.1) (*P* value: 8.42E−06)
	Conserved hypothetical protein (ADU40713) (*P* value: 0.00)	H. protein HPF16_0253 (YP_005778488) (*P* value: 0.0003)	H. protein HPF57_1344 (YP_005778058.1)* (*P* value: 0.003)
	H. protein KHP_0683 (ADU41063) (*P* value: 0.00)	H. protein HPKB_0212 (YP_005761745) (*P* value: 0.0003)	H. protein HPF57_0789 (YP_005777503.1) (*P* value: 1.22E−5)
	H. protein HPHPP23_1030 (ADU41054) (*P* value: 0.00)	H. protein HPF16_1167 (YP_005779402) (*P* value: 6.26E−21)	H. protein rpmJ (YP_005777967.1) (*P* value: 5.71E−05)
	H. protein HPKB_1436 (ADU40297) (*P* value: 0.00)	H. protein HPF16_0878 (YP_005779113 (*P* value: 2.48E−07)	H. protein HPF57_0703 (YP_005777417.1) (*P* value: 3.27E−09)
	H. protein HPF30_1072 (ADU40599) (*P* value: 0.00)	H. protein HPF16_1062 (YP_005779297) (*P* value: 1.34E−05)	H. protein HPF57_0549 (YP_005777263.1) (*P* value: 3.94E−07)
			H. protein HPF57_1078 (YP_005777792.1) (*P* value: 2.39E−06)

The area of Japan from where the strains were isolated, and the disease status of the individuals from whom the *H.pylori* strains were isolated is indicated in brackets (‘PUD’ signifies peptic ulcer disease, ‘Ga’ gastritis, ‘GC’ gastric cancer). References describing the respective disease status for each strain are as follows: 35a and 83 (Yoshio Yamaoka, Michael E. DeBakey Veterans Affairs Medical Center, Baylor College of Medicine, personal communication), F16 (27) and F57 (27). Those genes that were inferred as being under positive selection, with a value of Ka/Ks > 1, are listed. p values generated by each respective likelihood ratio test are shown; values of *P* = 0.00 represent the rounding of small values of p by the codeml program. Predicted membrane localized proteins (according to either Psortb or CELLO) are denoted with an asterisk. ‘H. protein’ denotes ‘hypothetical protein’. Genbank accession numbers are in brackets.

**Table 3.** Genes inferred as being under positive selection from the pairwise genome comparisons of four *H.pylori* strains isolated from Europe

B38 (France, ML)	G27 (Italy, PUD)	26695 (UK, Ga)	P12 (Germany, PUD)
Two-component response regulator (YP_003057199.1) (*P* value: 0.003)	2-oxoglutarate-acceptor oxidoreductase subunit OorD (YP_003057524.1) (*P* value: 0.0006)	trbI protein (NP_206843.1) (*P* value: 0.00)	sec-independent translocase (NP_207851.1) (*P* value: 0.037)
LPS 1,2-glycosyltransferase (YP_003056980.1) (*P* value: 0.001)	Diacylglycerol kinase (YP_003057423.1) (*P* value: 0.0003)	ABC transporter permease (YP_006934535) (*P* value: 0.00)	ABC transporter permease (YP_002301253) (*P* value: 0.023)
50S ribosomal protein L33 (YP_003057858.1) (*P* value: 0.00)	Haloacid dehalogenase (YP_003057862.1) (*P* value: 2.75E − 10)	Phosphotransacetylase (pta) (NP_207697.1) (*P* value: 0.010)	Molybdenum ABC transporter periplasmic molybdate-binding protein (modA) (NP_207271.1) (*P* value: 0.0001)
30S ribosomal protein S6 (YP_003057895.1) (*P* value: 0.004)	yceI protein (YP_003057934.1) (*P* value: 0.001)	Exodeoxyribonuclease VII small subunit (NP_208273.1) (*P* value: 0.009)	Exodeoxyribonuclease VII small subunit (YP_002302088.1) (*P* value: 0.00)
30S ribosomal protein L28 (YP_003057592) (*P* value: 0.001)	Flagellar biosynthesis protein (YP_003057130.1) (*P* value: 1.51E − 05)	30S ribosomal protein S11 (NP_208087.1) (*P* value: 0.0002)	tenA transcriptional regulator (NP_208079.1) (*P* value: 0.004)
CatIon transport subunit for cbb3-type oxidase (YP_003057819.1) (*P* value: 3.14E − 09)	sec-independent translocase (YP_003057182.1) (*P* value: 0.0007)	30S ribosomal protein S19 (NP_208107.1) (*P* value: 0.00)	Lipoprotein (NP_208229.1) (*P* value: 0.00)
Biopolymer transport protein ExbD (YP_003057987.1) (*P* value: 0.002)	Acetone carboxylase gamma subunit (YP_003057426.1) (*P* value: 0.0001)	Heat-inducible transcription repressor (NP_206911.1) (*P* value: 0.00)	Flagellar protein FlaG (NP_207544.1) (*P* value: 0.007)
Type IV restriction-modification enzyme (YP_003057166.1) (*P* value: 1.39E − 07)	rRNA large subunit methyltransferase (YP_003057653.1) (*P* value: 0.003)	Ribosome maturation factor rimP (NP_207836.1) (*P* value: 0.010)	NADH dehydrogenase subunit K (NP_208062.1) (*P* value: 0.03)
Dihydroneopterin aldolase (YP_003057321.1) (*P* value: 4.51E − 09)	Chorismate mutase PheA (YP_003057099.1) (*P* value: 0.0003)	Neuraminyllactose-binding hemagglutinin precursor (NLBH) (NP_207289.1) (*P* value: 0.00)	ATP-binding protein (NP_206967.1) (*P* value: 0.001)
RNA-binding protein (YP_003057806.1 (*P* value: 0.0001)	H. protein HELPY_1408 (YP_003058057.1) (*P* value: 0.002)	30S ribosomal protein S4 (NP_208086.1) (*P* value: 0.0002)	Pore-forming cytolysin (YP_003057160.1) (*P* value: 0.0002)
H. protein HELPY_1050 * (YP_003057739) (*P* value: 5.40E − 07)	H. protein HELPY_0637 (YP_003057396) (*P* value: 1.10E − 05)	Ribonuclease H (NP_207455) (*P* value: 0.001)	Ribonuclease H (YP_002301307) (*P* value: 0.001)
H. protein HELPY_0735 (YP_003057482) (*P* value: 0.003)	H. protein HELPY_0294* (YP_003057096.1) (*P* value: 0.002)	H. protein HP1405 (NP_207861) (*P* value: 0.001)	H. protein HP1405 (NP_208196) (*P* value:0.000)
H. protein HELPY_0206 (YP_003057019) (*P* value: 8.70E − 11)	H. protein HELPY_0581 (YP_003057349) (*P* value: 6.22E − 04)	H. protein HP0095 (NP_206895.1) (*P* value: 0.0001)	Chain C, crystal structure of Flis-Hp1076 complex in H. Pylori (NP_207867.1) (*P* value: 0.011)
H. protein HELPY_1408 (YP_003058057.1) (*P* value: 2.01E − 09)	H. protein HELPY_1405 (YP_003058054) (*P* value: 0.002)	H. protein HP1579* (NP_208370.1) (*P* value: 0.004)	H. protein HP1065* (ID: NP_207856.1) (*P* value: 0.03)
H. protein HELPY_0386 (YP_003057178) (*P* value: 7.60E − 05)	H. protein HELPY_0671 * (YP_003057425) (*P* value: 5.83E − 05)	H. protein HP0219 (NP_207017) (*P* value: 0.001)	H. protein HP0203 (NP_207002.1) (*P* value: 0.002)
H. protein HELPY_0261 (YP_003057065) (*P* value: 0.002)	H. protein HELPY_1039 (YP_003057728) (*P* value: 3.31E − 05)	H. protein HP79_07203 (NP_207845.1) (*P* value: 0.034)	H. protein HP0444 (NP_207242.1) (*P* value: 0.026)
	H. protein HELPY_0254 * (YP_003057060) (*P* value: 0.0005)		H. protein HP0716 (NP_207510.1) (*P* value: 0.01)
			H. protein HP0868 (NP_207662.1) (*P* value: 0.01)
			H. protein HP0350 (NP_207148.1) (*P* value: 0.02)
			H. protein HP0150 * (NP_206949.1) (*P* value: 0.023)
			H. protein HP0556 (NP_207856.1) (*P* value: 0.01)

The country of origin and disease status of the individuals from whom the *H.pylori* strains were isolated is indicated in brackets (‘ML’ signifies malt lymphoma, ‘PUD’ peptic ulcer disease, ‘Ga’ gastritis). References describing the respective disease statuses for each strain were B38 (58), G27 [59], 26695 [60] and P12 [61]. Those genes that were inferred as being under positive selection, with a value of Ka/Ks > 1, are listed. Predicted membrane localized proteins (according to either Psortb or CELLO) are denoted with an asterisk. ‘H. protein’ denotes ‘hypothetical protein’.

Of the genes identified in the analysis that were annotated, 38% had homologs with known roles in host adaptation and/or pathogenicity (Supplementary Material S1). Among these were genes involved in cell adhesion (CMP-N-acetylneuraminic acid synthetase, strain F57), secretion (sec-independent translocase strain G27 and P12), host recognition (neuraminyllactose-binding hemagglutinin, strain 35a) and host colonization (urease-enhancing factor and flgM protein, strain F16). These results indicate the efficacy of using an evolutionary rationale to identify potential strain-specific pathogenic factors. In addition, a number of genes were identified that have been proposed as drug targets, such as shikimate kinase (strain 83) [62], adenine-specific DNA methyltransferase (strain F16) [63], sialidases (strain F16) [64] and chorismate mutase (strain 83) [65]. While previously the *cagA* oncogene has been inferred to be under positive selection [66–70], this was not detected using our methodology. There are two potential explanations for this; firstly, our methodology uses Ka/Ks, which is a conservative measure. Second, *H.pylori* isolated from Amerindian populations show the strongest degree of inferred positive selection on *cagA* [69], and these were not included in our study.

While the function of the unannotated proteins inferred as being under positive selection is unknown, it is possible to predict whether these proteins are membrane localized. Predicted membrane localization is indicated in Tables 2 and 3. Out of 65 unannotated proteins inferred as being under positive selection, 13 were predicted to be membrane proteins. These are good candidates as novel pathogenic factors, given that many pathogenic factors are membrane localized, and may represent potential markers for gastric cancer. Table 4 shows genes inferred as being under positive selection that were identified in more than one pairwise comparison; these represent examples of convergent evolution, with inferred positive selection occurring in parallel in different strains. All these genes are located in the cytoplasm, with the exception of membrane localized sec-independent translocase, and participate in various cellular housekeeping functions. The genes are probably under similar selective pressures in the different human populations, and so are probably not greatly influenced by differences in host genetics or the stomach environment. Interestingly, sec-independent translocases are involved in secretion and so have a potential link to pathogenicity [71].

**Table 4.** Genes under inferred positive selection in more than one genome from the eight pairwise genome comparisons

Gene	Strains (gene accession number in brackets)	Biological process	Cellular localization
sec-independent translocase	G27 (YP_003057182.1), P12 (NP_207851.1)	Secretion	Plasma membrane
Urease-enhancing factor	F16 (YP_005779079), 35A (YP_005770181)	Metabolism	Cytoplasmic
ABC transporter permease	26695 (YP_006934535), P12 (YP_002301253)	Transport	Periplasmic
Ribonuclease H	26695 (NP_207455), P12 (YP_002301307)	RNA catabolic process	Cytoplasmic
Hypothetical protein HP1405	26695 (NP_208196.1), P12 (NP_208196)	Unknown	Cytoplasmic
Riboflavin synthase alpha	F16 (YP_005779723.1), F57 (YP_005778223.1)	Metabolism	Cytoplasmic
Chorismate mutase	G27 (YP_003057099.1), 83 (YP_005769657.1)	Metabolism	Cytoplasmic
Exodeoxyribonuclease VII small subunit	26695 (NP_208273.1), P12 (YP_002302088.1)	DNA repair	Cytoplasmic

The table shows those genes that are inferred as being under positive selection in more than one genome examined.

### Potential causes of differences in the strength of adaptive evolution between Japanese and European strains

The causes of lineage-specific variation in rates of adaptation are not fully understood. While Darwin proposed slow, gradualist evolutionary change, bursts of adaptation such as is observed in adaptive radiations are well known, as are examples of the contrasting case of evolutionary stasis. So, while it is clear that rates of adaptation may vary at the phenotypic level, on a genomic level this has been little explored. Here, we present an example of differential adaptive evolution between eight strains of *H.pylori*, which contrasts with their similarity in levels of purifying selection. Hence, the overall strength of adaptation, using the measure of number of genes inferred as being under positive selection, is markedly stronger in the Japanese strains. In prokaryotes, lineage-specific differences in genome wide adaptive pressure have also been reported in the *Streptococcus* [72] and *Campylobacter* [73] genera. The identities of the genes inferred as being under positive selection themselves are mostly different in the eight strains. This indicates lineage-specific differences in adaptive pressures. There could be three different explanations for these differences, which are not mutually exclusive;

1. Differences in the stomach environment unrelated to host genetics. Differences in the stomach environment may be linked to dietary factors, smoking, levels of exercise, and differences in microbiota [74] and are expected to be different in the populations examined.
2. Differences in the bacterial genetic background. The genetic background of the bacteria may exert an influence via epistatic effects, which may lead to differences in evolutionary trajectories [75]. Alternatively, specific bacterial factors may influence the overall immune response to the bacteria, thus increasing overall selection pressure
3. Differences in host genetics. This is discussed in the following section.

### Potential role of the immune system

Positive selection on microbial pathogenic factors often occurs via a selective pressure exerted by the host immune response [28], with antigenic moieties on the surface of the pathogenic factor undergoing adaptive evolution in order to avoid host immune recognition. Thus, greater levels of adaptive evolution in the Japanese strains may reflect an enhanced immune response and corresponding differences in host genetics. Strong differences in immune response, reflecting host genetics, is observed for a range of human pathogens, and differences in response may also be observed depending on the human population. In the case of *H.pylori*, polymorphisms in a number of immune genes have been shown to increase the immune response to infection and increase the risk of gastric cancer [76, a review]. While polymorphisms are often show differential distributions in different human populations, surprisingly few studies have focussed on population specific host genetic susceptibility factors to *H.pylori.* In one example, blood group, which shows strong populational biases, influences *H.pylori* infection via differential binding efficiency of *H.pylori* BabA protein to blood group antigens [77]. Host genetic background may also play a role; in one study IL-1B and IL-1 receptor antagonist gene polymorphisms were associated with gastric cancer risk in Caucasians but not Asians [78]. An elevated immune response would be expected to result in stronger selective pressure on the bacteria, which would be indicated by enhanced adaptive evolution of individual genes, given that only a subset of *H.pylori* genes are involved in interactions with the immune system [79]. This would occur via a more potent recognition of bacterial antigens, exerting a greater mortality on the bacterial population. The observation that 38% of the genes inferred as being under positive selection in the analysis have homologs involved in pathogenicity is consistent with this scenario.

This allows a hypothesis to be formulated that can explain both the elevated levels of adaptation observed in Japanese strains and the elevated levels of gastric cancer in Japanese populations. A heightened immune response leads to increased inflammation and this is turn may be linked to an elevated incidence of gastric cancer, given that inflammation has been identified as a key stage in its development [80]. There is a strong etiological link between *H.pylori* driven inflammation and gastric lymphoma [81], although the mechanistic link between inflammation and gastric carcinoma is not so clear [82]. Thus, a heightened immune response in the Japanese population may lead to both increased adaptation in *H.pylori* strains and increased oncogenesis. The strains examined in this analysis were isolated from individuals with a variety of different pathologies, but the signatures of positive selection that we detected are likely to have been accumulated over a longer time period than the lifespan of a single individual. More genome sequences will be required in order to fully test the hypothesis that elevated adaptation is related to increased inflammation, and the genes that are responsible for increased inflammation need to be established. A number of potential candidates have been identified in this analysis and experimental verification could be conducted on these.

### Relationship between transmission mode of *Helicobacter pylori* and pathogenicity

*Helicobacter pylori* is often regarded as vertically inherited, but increasing evidence shows that horizontal transmission may be more common than previously recognized, especially in developing countries [83–85]. The argument can be made that if *H.pylori* is entirely vertically transmitted, the observation of signatures of positive selection we detected in known and potential pathogenic factors of *H.pylori* is contradictory. This is because if the bacteria are vertically transmitted they are expected to evolve to minimize harmful effects [86], while if they are horizontally transmitted then they are expected to have a degree of pathogenicity [87]. Thus, our results are inconsistent with a strictly vertical mode of transmission. The age of transmission may be important; vertical transmission has been observed from mother to child, i.e. during reproductive age. This might help to explain the late onset of gastric cancer; it occurs after transmission has occurred and is consistent with the increasing evidence that *H.pylori* is beneficial, with pathogenic effects exerting themselves later in life [19].

## Conclusion

While there are similarities in the levels of purifying selection, Japanese *H.pylori* genomes have a greater number of genes inferred as being under positive selection than European strains and present new potential protein factors that interact with the host. These results help to further our understanding of the host interaction and pathogenesis of *H.pylori.* In particular, we propose that elevated levels of adaptation in these genomes may indicate an elevated immune response, and hypothesise that this provides a connection with elevated levels of gastric cancer in Japanese populations. In addition, in the absence of functionally annotated homologs, our procedure may have value in identifying potential novel pathogenic factors from pathogen genomes which could be crucial for adaptation to the host.

## SUPPLEMENTARY DATA

Supplementary data is available at *EMPH* online.

## FUNDING

This research was funded by the Biology Department, Faculty of Natural Sciences, UPR-Rio Piedras and supported by NSF equipment grant 0959864.

**Conflict of interest**: None declared.

## Supplementary Material

Supplementary Data
