# Supplementary material for: Elevated levels of adaption in Helicobacter pylori genomes from Japan; a link to higher incidences of gastric cancer?
Source: Evol Med Public Health. 2015 Mar 18;2015(1):88–105. doi: 10.1093/emph/eov005 (PMC4419197; doi:10.1093/emph/eov005)
Supplement: Supplementary Data [file supp_2015_1_88__index.html]

Elevated levels of adaption in Helicobacter pylori genomes from Japan; a link to higher incidences of gastric cancer? — Supplementary Data 

# Elevated levels of adaption in *Helicobacter pylori* genomes from Japan; a link to higher incidences of gastric cancer?

## Supplementary Data

files

**Files in this Data Supplement:**

- Supplementary Data - docx file
- Supplementary Data - tif file
- Supplementary Data - doc file
