# Supplementary material for: Elevated levels of adaption in Helicobacter pylori genomes from Japan; a link to higher incidences of gastric cancer?
Source: Evol Med Public Health. 2015 Mar 18;2015(1):88–105. doi: 10.1093/emph/eov005 (PMC4419197; doi:10.1093/emph/eov005)
Supplement: Supplementary Data [file supp_eov005_New_Microsoft_Office_Word_Document.docx]

**Supplementary Document 1**

Genes inferred as being under positive selection in the study and references in the literature to homologs involved in pathogenicity and host interaction.

**Supplementary Figure 1** **Multiple genome alignment of eight strains of *Helicobacter pylori*** **used in the pairwise genome comparisons**

Each horizontal panel represents one genome sequence. The scale shows genome coordinates in base pairs, and colored blocks indicate a genomic region that has been aligned in other strains that are connected by lines. Areas in white inside the blocks show the average conservation within corresponding genome regions (more white = less conservation). The alignment was performed using the progressive Mauve function in the MAUVE 2.3 package (Darling, A.C., Mau, B., Blattner, F.R., Pema, N.T. 2004. Mauve: multiple alignment of conserved genomic sequence with rearrangements. Genome Res. 14, 1394-1403.).
