# Supplementary figures and images for: Elevated levels of adaption in Helicobacter pylori genomes from Japan; a link to higher incidences of gastric cancer?
Source: Evol Med Public Health. 2015 Mar 18;2015(1):88–105. doi: 10.1093/emph/eov005 (PMC4419197; doi:10.1093/emph/eov005)

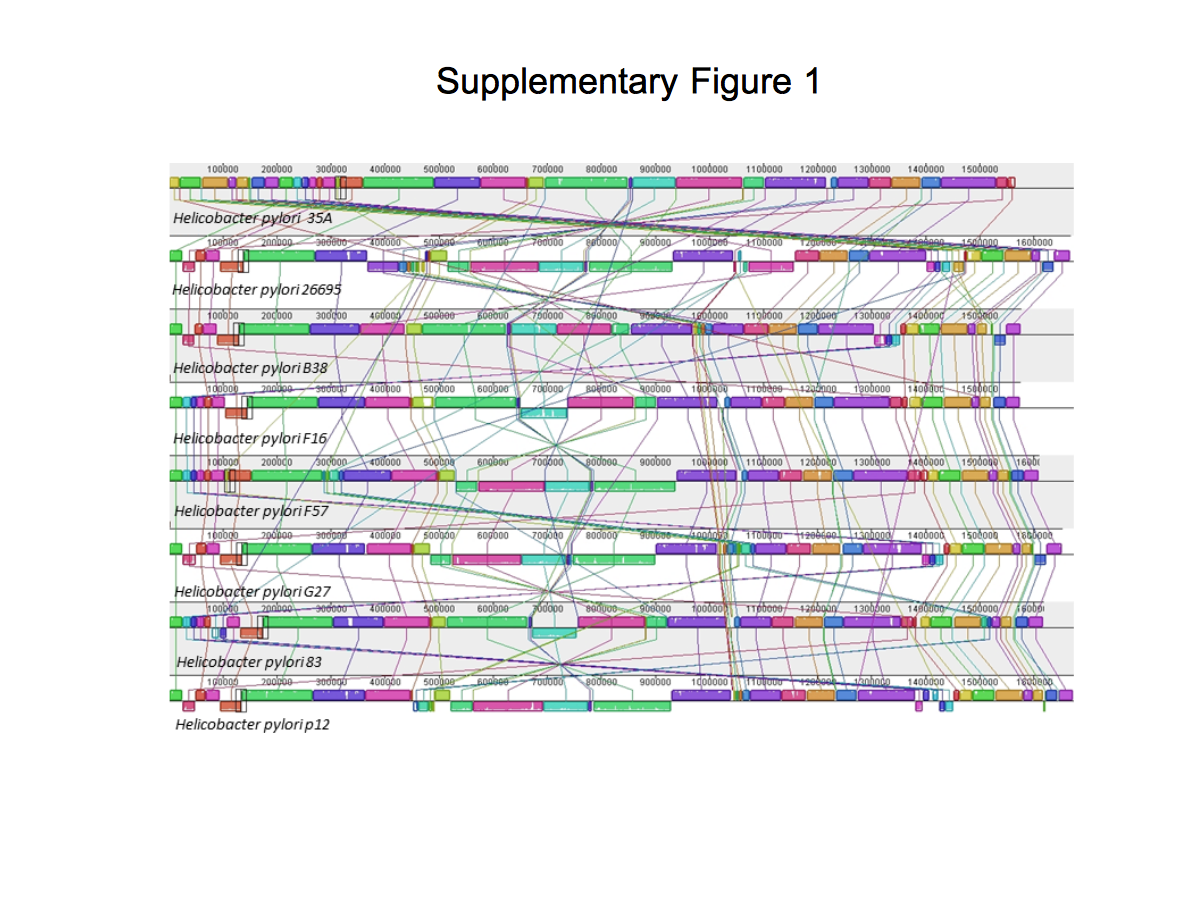

Supplement: Supplementary Data [file supp_eov005_suppfigure1title.tif]
