# Supplementary material for: Elevated levels of adaption in Helicobacter pylori genomes from Japan; a link to higher incidences of gastric cancer?
Source: Evol Med Public Health. 2015 Mar 18;2015(1):88–105. doi: 10.1093/emph/eov005 (PMC4419197; doi:10.1093/emph/eov005)
Supplement: Supplementary Data [file supp_eov005_Suppleme_Document1-checked.doc]

**Supplementary Document 1**

**Genes identified as being under positive selection with links in the literature to pathogenicity and host interaction**

**Japanese strain 35a**

Flagellar hook-associated protein 2, fliD (YP_005769952.1) and Flagellar hook-basal body complex protein FliE (YP_005769337) (Eaton et al., 1992, Moens and Vanderleyden, 1996)

Probable outer membrane protein (ADU41365.1) (Alm et al. 2000, Sugimoto et al. 2011)

Neuraminyllactose-binding hemagglutinin (YP_005769912.1) (Also known as Hpa; Cahlson et al., 2006)

Urease-enhancing factor (YP_005770181.1) (enhances activity of urease, a pathogenic factor; Eaton et al., 1991)

Pseudouridine synthase D (YP_005769708.1) (Ahn et al., 2004)

Disulfide interchange protein (YP_005770340.1) (Peek and Taylor, 1992)

Pyrroline-5-carboxylate reductase (YP_005770419) (Yang et al., 2006)

**Japanese Strain 83**

Proteobacterial sortase system OmpA family protein (YP_005769939.1) (Confer and Ayalew, 2012)

Polar flagellin (YP_005769953) (Eaton et al., 1992, Moens and Vanderleyden, 1996)

HOP family outer membrane porin (HOPE) (YP_005769995) (Peck et al 1999, Bauer and Meyer 2011)

Shikimate kinase (YP_005769533.1) (gene name aroK; Park et al. 2009, Schwager et al. 2012)

Chorismate mutase (YP_005769657.1) (Degrassi et al., 2010)

Lipid A 1-phosphatase (YP_005769383.1) (Coats et al., 2009)

Methyl-accepting chemotaxis protein (YP_005769452.1) (McLaughlin et al., 2012, Nishiyama et al., 2012)

Poly(A) polymerase (YP_005770052.1) (Altmeyer et al., 2010)

**Japanese Strain F16**

Urease-enhancing factor (YP_005770181.1) (enhances activity of urease, a pathogenic factor; Eaton et al., 1991)

cag pathogenicity island protein (CagG) (BAJ55413.1) (Saito et al., 2005)

flgM protein (BAJ55661.1) (Eaton et al., 1992, Moens and Vanderleyden, 1996)

Secreted protein involved in flagellar motility (YP_006219827) (Eaton et al., 1992, Moens and Vanderleyden, 1996)

Sialidase A (YP_003728769) (Corfield 1992)

Adenine specific DNA methyltransferase (BAJ54643.1) (Sun et al., 2010)

**Japanese strain 57**

Thioredoxin (BAJ55951.1) (Windle et al., 2000)

Outer membrane protein (BAJ55960.1) (Alm et al., 2000, Sugimoto et al. 2011)

CMP-N-acetylneuraminic acid synthetase (BAJ54930.1) (Eaton et al. 1992, Josenhans et al. 2002)

Putative peroxidase (BAJ56074.1) (Wang et al. 2005, Jittawuttipoka et al., 2009)

cag pathogenicity island protein (CagU) (BAJ55421) (As part of the cag PAI; Mobley 1996)

feoA gene product (YP_005770102) (Perry et al., 2007)

**European strain G27**

2-oxoglutarate-acceptor oxidoreductase subunit OorD (YP_003057524.1) (Hughes et al., 1998)

YceI protein (YP_003057934) (Sisinni et al., 2010, El-Halfawy and Valvano 2013)

Flagellar biosynthesis protein (YP_003057130) (Eaton et al., 1992, Moens and Vanderleyden, 1996)

Chorismate mutase PheA (YP_003057099.1) (Degrassi et al., 2010)

Haloacid dehalogenase (YP_003057861.1) (Tribble et al., 2006)

Diacylglycerol kinase (YP_003057423.1) (Shibata et al., 2009)

**European strain 26695**

trbI protein (NP_206843.1) (trbI is part of the tra operon, involved in transfer of plasmid transfer; Camacho and Casadesus 2002)

Neuraminyllactose-binding hemagglutinin precursor (NLBH) (NP_207289.1) (Chaturvedi et al., 2001)

Phosphotransacetylase (pta) (NP_207697.1) (Kim et al., 2006)

**European strain p12**

Lipoprotein (NP_208229.1) (Sutcliffe and Russell, 1995, Kovacs-Simon et al., 2011)

Flagellar protein FlaG (NP_207544.1) (Eaton et al., 1992, Moens and Vanderleyden, 1996)

Pore-forming cytolysin (YP_003057160.1) (Martino et al., 2001)

Chain C, Crystal Structure Of fliS-Hp1076 Complex (NP_207867.1) (Zhang et al., 2002)

**European strain B38**

LPS 1,2-glycosyltransferase (YP_003056980.1) (Moran 1995, Li and Wang 2012)

Biopolymer transport protein ExbD (YP_003057987.1) (Watson et al., 2005)

Two-component response regulator (YP_003057199.1) (Trihn et al., 2013)

**References**

Ahn, K.S., Ha, U., Jia, J., Wu, D., Jin, S. 2004. The *truA* gene of *Pseudomonas aeruginosa* is required for the expression of type III secretory genes. Microbiol. 150, 539-547.

Alm, RA; Bina, J; Andrews, BM; Doig, P; Hancock, RE; Trust, TJ. 2000. Comparative genomics *of Helicobacter pylori*:  analysis of the outer membrane protein families. Infect. Immunol. 68*,* 4155−4168.

Altmeyer, M., Barthel, M., Eberhard, M., Rehrauer, H., Hardt, W.D., Hottiger, M.O. 2010. Absence of poly(ADP-ribose) polymerase 1 delays the onset of *Salmonella enterica* serovar *Typhimurium*-induced gut inflammation. Infect Immun. 78, 3420-3431.

Bauer, B., Meyer, T.F. 2011. The human gastric pathogen *Helicobacter pylori* and its association with gastric cancer and ulcer disease. Ulcers. 340157.

Camacho, E.M., Casadesus, J. 2002. Conjugal transfer of the virulence plasmids of *Salmonella enterica* is regulated by the leucine-responsive regulatory protein and DNA adenine methylation. Mol Microbiol. 44, 1589-1598.

Carlsohn, E., Nyström, J., Bölin, I., Nilsson, C.L., Svennerholm, A.M. 2006. HpaA is essential for *Helicobacter pylori* colonization in mice. Infect Immun. 74, 920-926.

Chaturvedi, G., Tewari, R., Agnihotri, N., Vishwakarma, R.A., Ganguly N.K. 2001. Inhibition of *Helicobacter pylori* adherence by a peptide derived from neuraminyl lactose binding adhesin. Mol Cell Biochem. 228, 83-89.

Coats, S.R., Jones, J.W., Do, C.T., Braham, P.H., Bainbridge, B.W., To, T.T., Goodlett, D.R., Ernst, R.K., Darveau, R.P. 2009. Human Toll-like receptor 4 responses to *P. gingivalis* are regulated by lipid A 1- and 4'-phosphatase activities. Cell Microbiol. 11, 1587-1599.

Confer, A.W., Ayalew, S. 2012. The OmpA family of proteins: Roles in bacterial pathogenesis and immunity. Vet Microbiol. 163, 207-222.

Corfield, T. 1992. Bacterial sialidases – roles in pathogenicity and nutrition. Glycobiology. 2, 509-521.

Degrassi, G., Devescovi, G., Bigirimana, J., Venturi, V. 2010. *Xanthomonas oryzae* pv. oryzae XKK.12 contains an AroQgamma chorismate mutase that is involved in rice virulence. Phytopathology. 100, 262-270.

Eaton, K.A., Brooks, C.L., Morgan, D.R., Krakowka, S. 1991. Essential role of urease in pathogenesis of gastritis induced by Helicobacter pylori in gnotobiotic piglets. Infect Immun. 59, 2470–2475.

Eaton, K. A., D. R. Morgan, and S. Krakowka. 1992. Motility as a factor in the colonisation of gnotobiotic piglets by *Helicobacter pylori*. J. Med. Microbiol. 37, 123-127.

El-Halfawy, O.M., Valvano, M.A. 2013. Chemical communication of antibiotic resistance by a highly resistant subpopulation of bacterial cells. PLoS One. 8, e68874.

Hughes, N.J., Clayton, C.L., Chalk, P.A., Kelly, D.J. 1998. *Helicobacter pylori* porCDAB and oorDABC genes encode distinct pyruvate: flavodoxin and 2-oxoglutarate:acceptor oxidoreductases which mediate electron transport to NADP. J Bacteriol. 180, 1119-128.

Jittawuttipoka, T., Buranajitpakorn, S., Vattanaviboon, P., Mongkolsuk, S. 2009. The catalase-peroxidase KatG is required for virulence of *Xanthomonas campestris* pv. campestris in a host plant by providing protection against low levels of H_2_O_2_. J Bacteriol. 191, 7372-7377.

Josenhans, C., Vossebein, L., Friedrich, S., Suerbaum, S. 2002. The neuA/flmD gene cluster of *Helicobacter pylori* is involved in flagellar biosynthesis and flagellin glycosylation. FEMS Microbiol. Lett. 7, 165-172.

Kim, Y.R., Brinsmade, S.R., Yang, Z., Escalante-Semerena, J., Fierer, J. 2006. Mutation of phosphotransacetylase but not isocitrate lyase reduces the virulence of *Salmonella enterica* serovar *Typhimurium* in mice. Infect Immun. 74, 2498-2502.

Kovacs-Simon, A., Titball, R.W., Michell, S.L. 2011. Lipoproteins of bacterial pathogens. Infect Immun. 79, 548-561.

Li, J., Wang, N. 2012. The *gpsX* gene encoding a glycosyltransferase is important for polysaccharide production and required for full virulence in *Xanthomonas citri* subsp. *citri*. BMC Microbiol. 12, 31.

Martino, M.C., Stabler, R.A., Zhang, Z.W., Farthing, M.J., Wren, B.W., Dorrell, N. 2001. *Helicobacter pylori* pore-forming cytolysin orthologue TlyA possesses in vitro hemolytic activity and has a role in colonization of the gastric mucosa. Infect Immun. 69, 1697-703.

McLaughlin, H.P., Caly, D.L., McCarthy, Y., Ryan, R.P., Dow, J.M. 2012. An orphan chemotaxis sensor regulates virulence and antibiotic tolerance in the human pathogen *Pseudomonas* *aeruginosa*. PLoS One 7, e42205.

Mobley, H.L. 1996. Defining *Helicobacter pylori* as a pathogen: strain heterogeneity and virulence. Am J Med. 100, 2S-9S.

Moens, S., Vanderleyden, J. 1996. Functions of bacterial flagella. Crit. Rev. Microbiol. 22, 67–100.

Moran, A. P. 1995. Structure-bioactivity relationships of bacterial endotoxins. J. Toxicol. Toxin Rev. 14, 47–83.

Nishiyama, S., Suzuki, D., Itoh, Y., Suzuki, K., Tajima, H., Hyakutake, A., Homma, M., Butler-Wu, S.M., Camilli, A., Kawagishi, I. 2012. Mlp24(McpX) of *Vibrio cholerae* implicated in pathogenicity functions as a chemoreceptor for multiple amino acids. Infect Immun. 80, 3170-3178.

Park, Y-J., Song, E-S., Noh, T-H., Kim, H., Yang, K-S., Hahn, J-H., Kang, H-W., Lee, B-M. 2009. Virulence analysis and gene expression profiling of the pigment – deficient mutant of *Xanthomonas oryzae* pathovar *oryzae*. FEMS Microbiology Lett. 301, 149-155.

Peck, B., Ortkamp, M., Diehl, K.D., Hundt, E., Knapp, B. 1999. Conservation, localization and expression of HopZ, a protein involved in adhesion of *Helicobacter pylori*. Nuc Acids Res. 27, 3325-3333.

Peek, J.A., Taylor, R.K. 1992. Characterization of a periplasmic thiol:disulfide interchange protein required for the functional maturation of secreted virulence factors of *Vibrio cholerae*. Proc Natl Acad Sci USA. 89, 6210-6214.

Perry, R.D., Mier, I., Fetherston, J.D. 2007. Roles of the Yfe and Feo transporters of *Yersinia* *pestis* in iron uptake and intracellular growth. Biometals. 20, 699-703.

Saito, H., Yamaoka, Y., Ishizone, S., Maruta, F., Sugiyama, A., Graham, D.Y., Yamauchi, K., Ota, H., Miyagawa, S. 2005. Roles of *virD4* and *cagG* genes in the *cag* pathogenicity island of *Helicobacter pylori* using a Mongolian gerbil model. Gut. 54, 584-590.

Schwager, S., Agnoli, K., Kothe, M., Feldmann, F., Givskov, M., Carlier, A., Eberl, L. 2012. Identification of *Burkholderia cenocepacia* Strain H111 virulence factors using nonmammalian infection hosts. Infect. Immun. 81, 143-153.

Shibata, Y., van der Ploeg, J.R., Kozuki, T., Shirai, Y., Saito, N., Kawada-Matsuo, M., Takeshita, T., Yamashita, Y. 2009. Kinase activity of the *dgk* gene product is involved in the virulence of *Streptococcus mutans*. Microbiology. 155, 557-565.

Sisinni, L., Cendron, L., Favaro, G., Zanotti, G. 2010. *Helicobacter pylori* acidic stress response factor HP1286 is a YceI homolog with new binding specificity. FEBS J. 8, 1896-1905.

Sugimoto, M., Ohno, T., Graham, D.Y., Yamaoka, Y. 2011. *Helicobacter pylori* outer membrane proteins on gastric mucosal interleukin 6 and 11 expression in Mongolian gerbils. J Gastroenterol Hepatol. 26, 1677-1684.

Sun, K., Jiao, X.D., Zhang, M., Sun, L. 2010. DNA adenine methylase is involved in the pathogenesis of *Edwardsiella tarda*. Vet Microbiol. 141, 149-154.

Sutcliffe, IC, Russell, RR, 1995. Lipoproteins of gram-positive bacteria. J. Bacteriol. 177, 1123-1128.

Tribble, G.D., Mao, S., James, C.E., Lamont, R.J. 2006. A *Porphyromonas gingivalis* haloacid dehalogenase family phosphatase interacts with human phosphoproteins and is important for invasions. Proc Natl Acad Sci USA. 103, 11027-11032.

Trihn, M., Ge, X., Dobson, A., Kitten, T., Munro, C.L., Xu, P. 2013. Two-component system response regulators involved in virulence of *Streptococcus pneumoniae* TIGR4 in infective endocarditis. PLoS One. 8, e54320.

Wang, G., Olczak, A.A., Walton, J.P., Maier, R.J. 2005. Contribution of the *Helicobacter pylori* thiol peroxidase bacterioferritin comigratory protein to oxidative stress resistance and host colonization. Infect Immun. 73, 378-384.

Watson, R.J., Joyce, S.A., Spencer, G.V., Clarke, D.J. 2005. The *exbD* gene of *Photorhabdus temperata* is required for full virulence in insects and symbiosis with the nematode *Heterorhabditis*. Mol Microbiol. 56, 763-773.

Windle, H.J., Fox, A., Ni Eidhin, D., Kelleher, D. 2000. The thioredoxin system of *Helicobacter pylori*. J Biol Chem. 275, 5081-5089.

Yang, Y., Xu, S., Zhang, M., Jin, R., Zhang, L., Bao, J., Wang, H. 2006. Purification and characterization of a functionally active *Mycobacterium tuberculosis* pyrroline-5-carboxylate reductase. Protein Expr Purif. 45, 241-248.

Zhang, Z.W., Dorrell, N., Wren, B.W., Farthingt, M.J. 2002. *Helicobacter pylori* adherence to gastric epithelial cells: a role for non-adhesin virulence genes. J Med Microbiol. 51, 495-502.
